# Low-Cost BD/MEMS Tightly-Coupled Pedestrian Navigation Algorithm

**DOI:** 10.3390/mi7050091

**Published:** 2016-05-16

**Authors:** Tianyu Lin, Zhenyuan Zhang, Zengshan Tian, Mu Zhou

**Affiliations:** Chongqing Key Lab of Mobile Communications Technology, Chongqing University of Posts and Telecommunications, Chongqing 400065, China; zhangzhenyuan163@163.com (Z.Z.); tianzs@cqupt.edu.cn (Z.T.); zhoumu@cqupt.edu.cn (M.Z.)

**Keywords:** BD/MEMS navigation, tight coupling, pedestrian dead reckoning, extended Kalman filter, low cost

## Abstract

Pedestrian Dead Reckoning (PDR) by combining the Inertial Measurement Unit (IMU) and magnetometer is an independent navigation approach based on multiple sensors. Since the inertial component error is significantly determined by the parameters of navigation equations, the navigation precision may deteriorate with time, which is inappropriate for long-time navigation. Although the BeiDou (BD) navigation system can provide high navigation precision in most scenarios, the signal from satellites is easily degraded because of buildings or thick foliage. To solve this problem, a tightly-coupled BD/MEMS (Micro-Electro-Mechanical Systems) integration algorithm is proposed in this paper, and a prototype was built for implementing the integrated system. The extensive experiments prove that the BD/MEMS system performs well in different environments, such as an open sky environment and a playground surrounded by trees and thick foliage. The proposed algorithm is able to provide continuous and reliable positioning service for pedestrian outdoors and thereby has wide practical application.

## 1. Introduction

With the rapid development of sensor technology, Location-Based Services (LBSs) bring much convenience to people’s daily lives [1], such as making friends, sharing locations, gathering the neighboring business information and planning walk paths [2,3]. LBSs also play an important role in military use and national security, as well as searching and rescuing actions in disasters.

The Global Navigation Satellite System (GNSS) is the most popular outdoor navigation system, which features global coverage. It brings huge economic and social benefits, like transportation, fishery, hydrologic monitoring, weather forecast and vehicle navigation [4]. However, its accuracy may decline rapidly when the signal from satellites is blocked by buildings and trees [5].

Micro-Electro-Mechanical Systems (MEMS) have the advantages of small size, low cost and integration ability [6]. Although the Inertial Navigation System (INS) can provide accurate relative navigation information and guarantee independent and strong anti-interference capacity, it was generally restricted in the military and aerospace industry before MEMS were presented due to the cost and government restrictions [7]. MEMS technology makes it possible to design low-cost, small-sized and light-weight INS for commercial use. MEMS-based INS calculates position, velocity and attitude by combining the data from various kinds of sensors, e.g., accelerometer, gyroscope and magnetometer. In the walking scenario, pedestrian dead reckoning (PDR) based on the pedestrian motion model is used to calculate the stride length of a pedestrian. However, the MEMS-INS suffers from error accumulation, which may cause large positioning error.

Based on the previous discussion, the GNSS is time independent, but performs poorly when the signal is degraded or there are less than four visible satellites, while the INS is environment independent, but constrained by error accumulation. To solve this problem, various kinds of integration approaches have been proposed to integrate the satellites and inertial sensors with the purpose of precise and continuous navigation [8]. The existing approaches can be classified into three categories, the loosely-coupled, tightly-coupled and deep tightly-coupled approaches. For the tightly-coupled approach, it can work properly when the number of visible satellites is less than four since the pseudo-range and pseudo-range rate are used [9]. In this paper, we propose a low-cost BD/MEMS tightly-coupled algorithm for pedestrian navigation, and meanwhile, a portable hardware platform is developed.

The rest of this paper is organized as follows. Some related work on the localization by the GNSS and INS is discussed in Section 2. In Section 3, we describe the proposed system. The experimental results are provided in Section 4. Finally, in Section 5, we conclude the paper.

## 2. Related Work

Transit navigation satellite system is one of the early representative satellite navigation systems. From 1960 to 1996, more than 30 transit navigation satellites were launched. However, the time cost for each positioning is significantly high, and the positioning accuracy is poor. After that, GPS and GLONASS were put into use in the 1990s. Nowadays, many advanced technologies are used in the latest satellite navigation systems, such as Binary Offset Carrier (BOC). BOC is used to improve the precision of orientation estimation and to enhance the anti-jamming and weak signal detection capacity [10,11,12,13,14].

The MEMS-based inertial sensors have been widely used due to the low cost [15]. To improve MEMS-INS performance, Vitanov in [16] proposed a Gaussian process-enhanced unscented Kalman filter architecture to perform the fault detection and isolation on gyros and accelerometers of strap-down MEMS-based INS. Aggarwal in [17] established a thermal model for the low-cost MEMS-based INS, which can be used for integrated vehicle navigation. Akeila in [18] proposed an error resetting approach using three accelerometers and three magnetometers for moving objects.

There are a large number of researchers focusing on the integration of GNSS and MEMS-based INS. This scheme guarantees meter-level localization accuracy when the GNSS performances are poor for the location services because of weak signals or less than four visible satellites. Angrisano in [19] integrated GPS/GLONASS with the low-cost MEMS-INS for pedestrian and vehicular navigation. Zhuang in [20] proposed an integrated navigation system by using a smartphone platform, which consists of a three-axis accelerometer, a three-axis gyroscope, a three-axis magnetometer and a GPS receiver. In the system, the loosely-coupled scheme based on the extended Kalman filter is implemented. Jia in [21] developed an integrated navigation system based on the low-cost MEMS-IMU and GPS receiver. This system is able to achieve continuous navigation, but the data information from the GPS and IMU is not well used. To address this problem, the tightly-coupled approach was considered recently. Lachapelle in [22] invented the tightly-coupled navigation system based on GPS and IMU to improve the pedestrian positioning accuracy when the signal from satellites is severely degraded. In [23], an improved INS/GPS integration routine using the unscented Kalman filter and the adaptive unscented Kalman filter was presented. It is concluded that UKF provides only a slight improvement in the navigation performance over EKF. In [24], a closely-coupled GPS/INS integration is described in which inertial measurements are combined with available GPS ranges even when less than four satellites are in view. The map-matching approach, which can be used to enhance the performance of the integrated GPS/MEMS system, is addressed in [25]. Godha in [26,27] compared the performance of the Personal Navigation System (PNS), GPS and the integrated PDR/GPS system. O’Keefe in [28] designed a navigation system by integrating the Ultra-Wideband (UWB) and satellite navigation system and meanwhile applied it to intelligent transportation.

Different from the previous literature, to take advantage of the BD with high positioning accuracy and MEMS-based INS with a low cost, we design a new tightly-coupled BD/MEMS system for pedestrian navigation.

## 3. System Description

**Figure 1:**
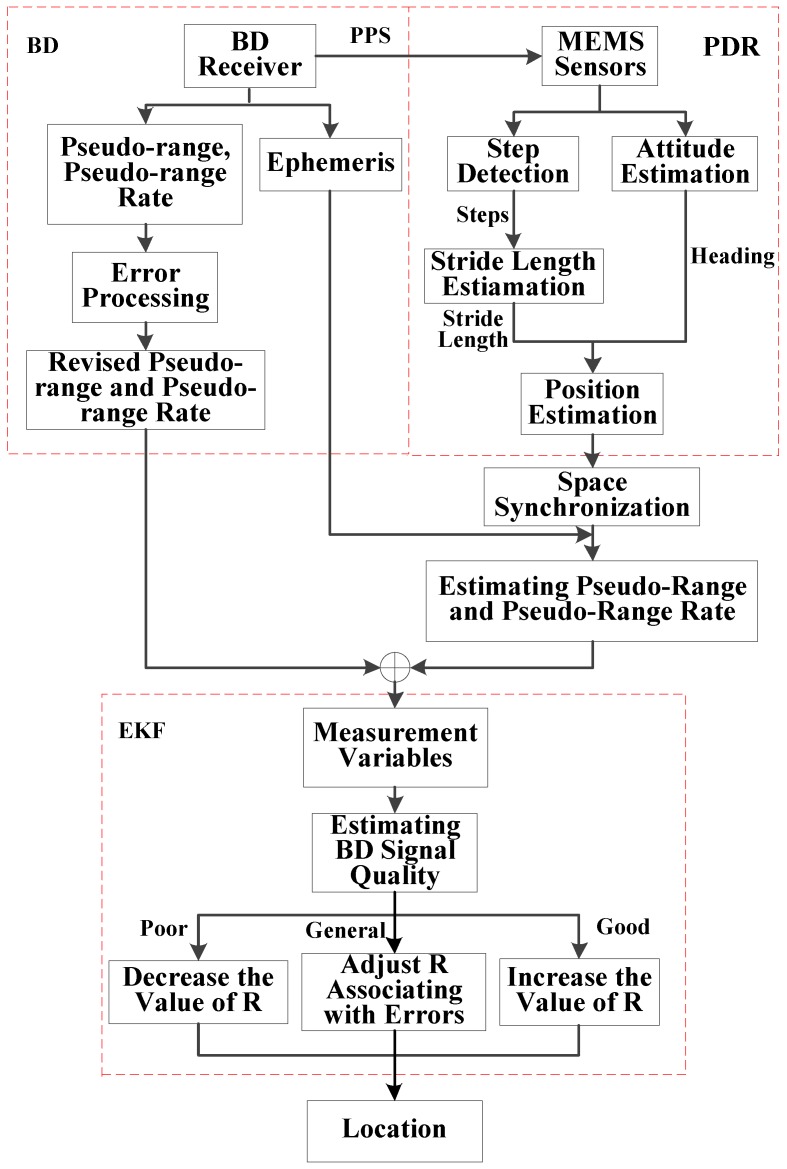
Architecture of the proposed system.

The flow chart of the proposed system is shown in Figure 1. The BD receiver provides the observed pseudo-range, pseudo-range rate and ephemeris information of satellites. At the same time, the pseudo-range and pseudo-range rate can be estimated by using the PDR and the ephemeris information of satellites. The differences between the observed and predicted pseudo-range and pseudo-range rate are selected as the measurement of Extended Kalman Filter (EKF). The estimation of the optimal locations and velocity can be obtained by adaptively adjusting the observation noise matrix of EKF according to the performance of BD and PDR.

### 3.1. Pedestrian Dead Reckoning

**Figure 2:**
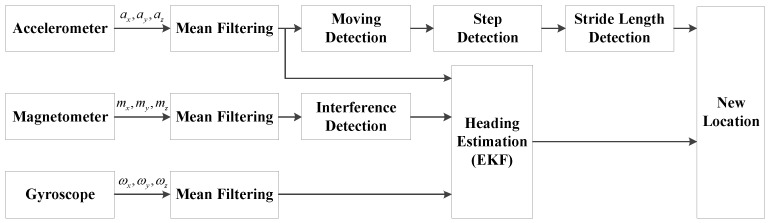
Flow chart of PDR.

PDR consists of three modules, step detection, stride length estimation and heading estimation. The basic idea of PDR is that each location is estimated based on the previous location, moving distance and heading. The flow chart of PDR is shown in Figure 2.

In the PDR system, all of the raw sensor data are first smoothed by using a mean filter to weaken noise. The modulus of three-axis acceleration is calculated and then used in step detection and stride length detection. In addition, the EKF is utilized to estimate the heading by integrating the data from the accelerometer, magnetometer and gyroscope.

#### 3.1.1. Step Detection

**Figure 3:**
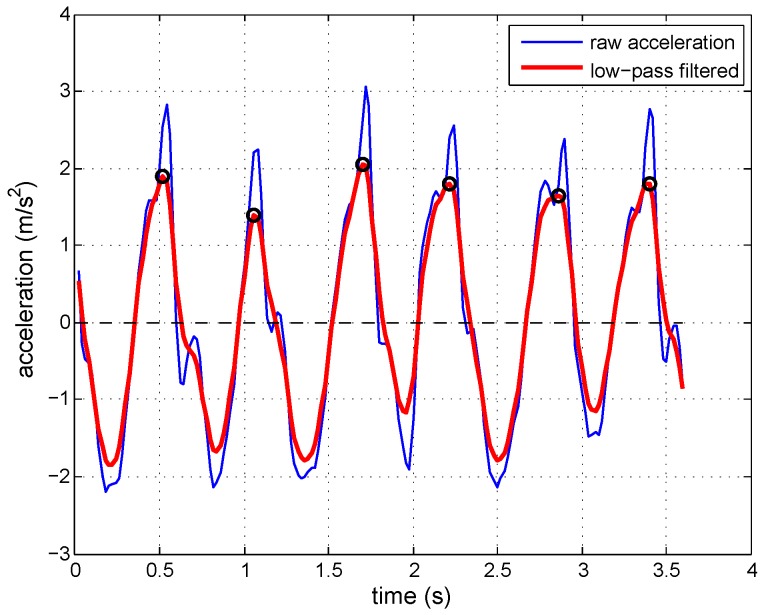
Acceleration data before and after filtering.

By analyzing the walking characteristics of a pedestrian and acceleration data, it can be found that the acceleration changes regular, like a sine wave. There will be an acceleration peak for each step. Therefore, the modulus of three-axis acceleration is used for step detection, and it can be obtained by:

(1) $$a_{i}^{total}=\sqrt{{\left(a_{i}^{x}\right)}^{2}+{\left(a_{i}^{y}\right)}^{2}+{\left(a_{i}^{z}\right)}^{2}}-g$$

where *g* is the gravitational acceleration and $a_{i}^{x},a_{i}^{y}$ and $a_{i}^{z}$ are the values of the tri-axis accelerometer of the *i*-th sample (with the sampling rate of 50 Hz). Due to the interference of sensor noise and hand shaking, the acceleration modulus is smoothed by using a low-pass filter before step detection. Figure 3 compares the acceleration data before and after filtering. It can be found that the multi-peak is removed, and this makes step detection more accurate. The black circles shown in the figure indicate the steps counted.

**Figure 4:**
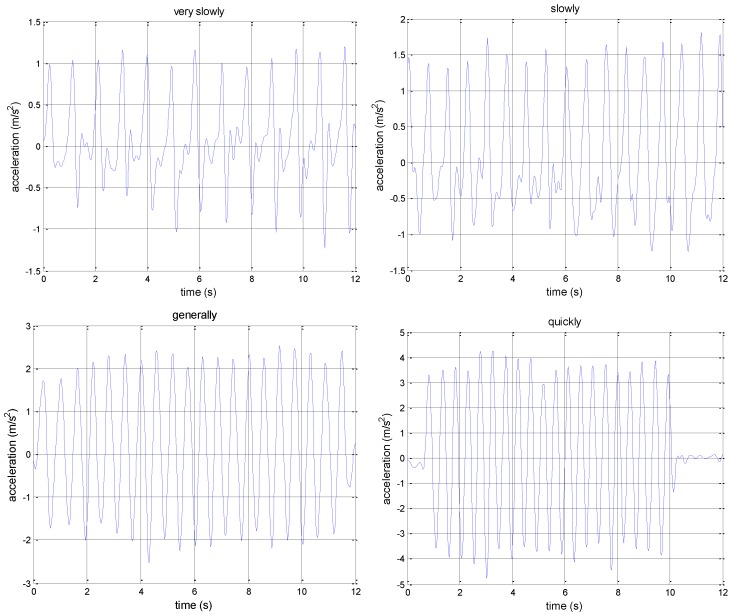
Acceleration modulus of different walking speeds.

Although both the peak and the trough can be detected, we found that peak detection is a better scheme by analyzing acceleration data at different walking speeds. Figure 4 shows the difference of the acceleration modulus of different walking speeds after low-pass filtering. The absolute value of the acceleration peak is larger than the trough. The peaks are more regular. By analyzing the different walking speed data, we set a proper threshold for detecting the real peak caused by pedestrian walking. It is 0.8 m/s${}^{2}$. In addition, the time between two adjacent peaks should not be too short. One cannot generally walk three steps in one second. The time threshold of two adjacent peaks is set as 16 samples (the sampling rate is 50 Hz). Using peak detection and the two thresholds, test results show that only one or two steps will not be detected.

#### 3.1.2. Stride Length Estimation

The empirical stride length model was proposed in [29] and verified in [30]. The previous work used vertical acceleration to estimate the stride length. However, considering that when there is one step forward, one’s body is accelerated uphill, the modulus of tri-axis acceleration is used in our paper. The stride length formula is shown as follows:

(2) $$stride\_length=c\sqrt[{4}]{a_{\max}-a_{\min}}$$

where $a_{\max}$ and $a_{\min}$ are the maximum and minimum of acceleration for each step and *c* is a coefficient that depends on each pedestrian. In previous work, the coefficient was determined through calibrations when someone first uses the model to estimate his or her stride length. The general method for estimating stride length is needed. This paper presented a modified method to make the empirical stride length model general by applying a Back Propagation (BP) neural network. The *c* is adaptive to different people and different walking speeds.

**Figure 5:**
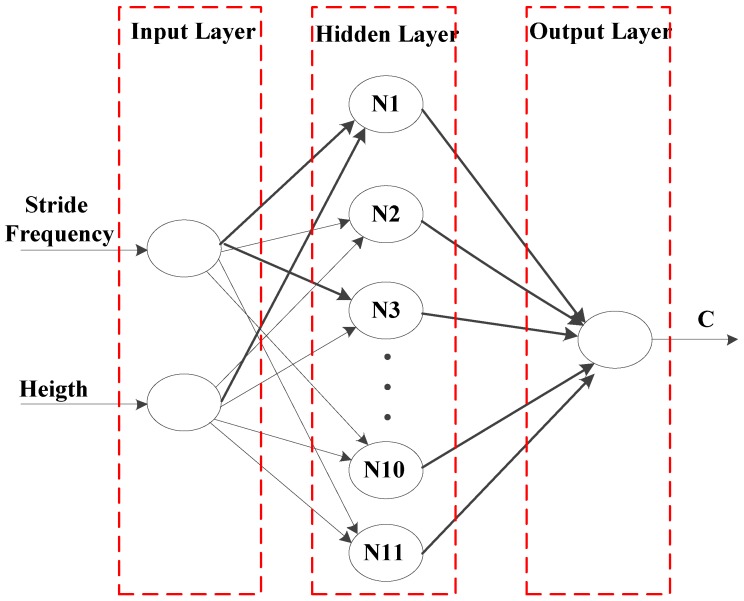
Topological structure of the BP neural network.

People’s height and walking frequency are related to their stride length [31]. Therefore, height and walking frequency were selected as the input of BP and *c* as the output. A neural network with one hidden layer was designed to adaptively adjust *c* in a real-time manner. According to the Kolmogorov theorem, the number of neurons in the hidden layer of a BP neural network, *l*, is constrained by:

(3) l>2m+1

where *m* is the number of input layer neurons. In addition, the value *l* can be obtained by the empirical formula [32]:

(4) $$l=\sqrt{m+n}+a$$

where *n* is the number of neurons in the output layer of the BP neural network and *a* is a constant in the range of [0, 10]. The above Equations (3) and (4) are only for reference; the specific node number depends on the training effect. According to our previous work, the value *l* was set as 11. The structure of the BP neural network is shown in Figure 5.

Then acceleration data were collected for BP training. After that, the BP is used to predict *c* for each step, and the stride length can be easily calculated by Equation (2).

#### 3.1.3. Attitude Estimation

**Figure 6:**
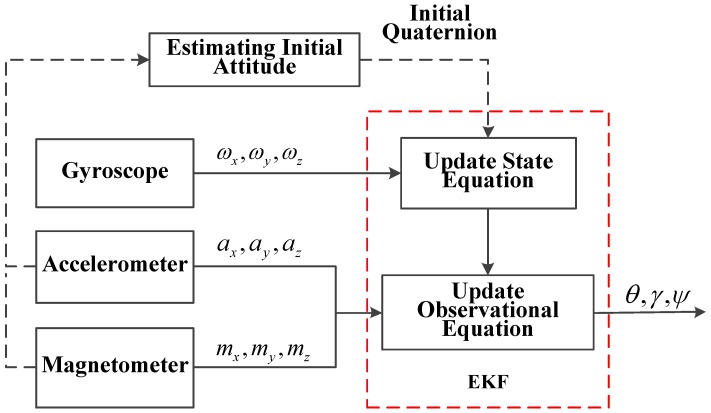
Flow chart of the attitude estimation algorithm.

The gyroscope easily suffers from error accumulation, while the heading of the pedestrian that is estimated by measuring the magnetic strength is normally stable. Based on this, we estimate the attitude of the pedestrian by using the EKF to integrate the data from the gyroscope, magnetometer and accelerometer. The flow chart of the proposed attitude estimation algorithm is shown in Figure 6.

By assuming that the pedestrian is static at the beginning, the vector of tri-axis acceleration values, $a_{x}$, $a_{y}$ and $a_{z}$, in the Body Frame System (BFS) is defined as:

(5) $$a^{b}={\left[\begin{array}{ccc} a_{x} & a_{y} & a_{z} \end{array}\right]}^{T}$$

Since the vector of tri-axis acceleration values under the North-East-Up (NEU) system is $a^{n}=[\begin{array}{ccc} 0 & 0 & g \end{array}{]}^{T}$, the relationship between these two vectors can be described as:

(6) $$\left[\begin{array}{c} a_{x}\\ a_{y}\\ a_{z} \end{array}\right]=C_{n}^{b}\left[\begin{array}{c} 0\\ 0\\ g \end{array}\right]={\left(C_{b}^{n}\right)}^{-1}\left[\begin{array}{c} 0\\ 0\\ g \end{array}\right]$$

where $C_{n}^{b}$ is the rotation matrix from NEU system to BFS, as shown in Equation (7). Since the acceleration values are independent of heading estimation, we set the initial heading value as ψ=0.

(7) $$C_{n}^{b}=\left(\begin{array}{ccc} \cos\gamma & 0 & \sin\gamma \\ \sin\theta \sin\gamma & \cos\theta & -\sin\theta \cos\gamma \\ -\cos\theta \sin\gamma & \sin\theta & \cos\theta \cos\gamma \end{array}\right)$$

where *θ* and *γ* are the pitch and roll angles, respectively. Then, we convert Equation (6) into:

(8) $$\left[\begin{array}{c} a_{x}\\ a_{y}\\ a_{z} \end{array}\right]=\left(\begin{array}{ccc} \cos\gamma & 0 & \sin\gamma \\ \sin\theta \sin\gamma & \cos\theta & -\sin\theta \cos\gamma \\ -\cos\theta \sin\gamma & \sin\theta & \cos\theta \cos\gamma \end{array}\right)\left[\begin{array}{c} 0\\ 0\\ g \end{array}\right]=\left[\begin{array}{c} \sin\gamma \\ -\sin\theta \cos\gamma \\ \cos\theta \cos\gamma \end{array}\right]g$$

Based on Equation (8), we obtain:

(9) $$\left\{\begin{array}{c} \gamma =\arctan\left(\frac{a_{x}}{\sqrt{a_{y}^{2}+a_{z}^{2}}}\right)\\ \theta =\arctan\left(-\frac{a_{y}}{a_{z}}\right) \end{array}\right.$$

Therefore, the relationship of the magnetic strengths under different frame systems is described as:

(10) $$\left\{\begin{array}{c} m_{x}^{n}=m_{x}^{b}\cos\gamma +m_{y}^{b}\sin\gamma \sin\theta -m_{z}^{b}\cos\theta \sin\gamma \\ m_{y}^{n}=m_{y}^{b}\cos\theta +m_{z}^{b}\sin\theta \end{array}\right.$$

where $m_{x}^{b},m_{y}^{b}$ and $m_{z}^{b}$ are magnetic strengths measured by the magnetometer under the BFS, while $m_{x}^{n}$ and $m_{y}^{n}$ are the ones under the NEU system. Since the heading estimation is determined by the east and north components of magnetic strength, $m_{x}^{n}$ and $m_{y}^{n}$, the heading can be estimated by:

(11) $$\psi =\arctan\left(\frac{m_{x}^{n}}{m_{y}^{n}}\right)$$

To estimate the attitude, we select the four quaternion elements as the state variables, as shown in Equation (12).

(12) $$X={\left[\begin{array}{cccc} q_{0} & q_{1} & q_{2} & q_{3} \end{array}\right]}^{T}$$

According to the quaternion principle of the strapdown inertial navigation system, the state equation is defined as:

(13) $$\dot{X}=\frac{1}{2}\left[\begin{array}{cccc} 0 & -\left(\omega_{x}-\varepsilon_{x}\right) & -\left(\omega_{y}-\varepsilon_{y}\right) & -\left(\omega_{z}-\varepsilon_{z}\right)\\ \left(\omega_{x}-\varepsilon_{x}\right) & 0 & \left(\omega_{z}-\varepsilon_{z}\right) & -\left(\omega_{y}-\varepsilon_{y}\right)\\ \left(\omega_{y}-\varepsilon_{y}\right) & -\left(\omega_{z}-\varepsilon_{z}\right) & 0 & \left(\omega_{x}-\varepsilon_{x}\right)\\ \left(\omega_{z}-\varepsilon_{z}\right) & \left(\omega_{y}-\varepsilon_{y}\right) & -\left(\omega_{x}-\varepsilon_{x}\right) & 0 \end{array}\right]X$$

where $\omega_{x},\omega_{y},\omega_{z}$ are the output values of the gyroscope and $\varepsilon_{x},\varepsilon_{y},\varepsilon_{z}$ are the errors in three different directions.

In addition, the acceleration values and magnetic strengths measured by the accelerometer and magnetometer respectively are selected as the measurement variables [33], as shown in Equation (14).

(14) $$Z={\left[\begin{array}{cccccc} a_{x} & a_{y} & a_{z} & m_{x} & m_{y} & m_{z} \end{array}\right]}^{T}$$

where $a_{x},a_{y}$ and $a_{z}$ are the normalized acceleration values, while $m_{x},m_{y}$ and $m_{z}$ are the normalized magnetic strengths in three different directions under the BFS. By using the EKF to estimate the optimal quaternion elements, the attitude can be estimated by:

(15) $$\begin{array}{c} \theta =\arctan\left(-\frac{2\left(q_{2}q_{3}+q_{0}q_{1}\right)}{q_{0}^{2}-q_{1}^{2}-q_{2}^{2}+q_{3}^{2}}\right)\\ \gamma =\arcsin\left(2\left(q_{1}q_{3}-q_{0}q_{2}\right)\right)\\ \psi =\arctan\left(-\frac{2\left(q_{1}q_{2}+q_{0}q_{3}\right)}{q_{0}^{2}+q_{1}^{2}-q_{2}^{2}-q_{3}^{2}}\right) \end{array}$$

#### 3.1.4. Position Estimation

Finally, the current position of pedestrian under the NEU system is estimated by:

(16) $$\left\{\begin{array}{c} N_{n}=N_{n-1}+d_{n-1}\cos\theta_{n-1}\\ E_{n}=E_{n-1}+d_{n-1}\sin\theta_{n-1} \end{array}\right.$$

where $\left(E_{n-1},N_{n-1}\right)$ is the previous position of the pedestrian and $d_{n-1}$ and $\theta_{n-1}$ are the stride length and heading value at the previous timestamp.

### 3.2. Data Synchronization

#### 3.2.1. Space Synchronization

To achieve the space synchronization of the data under the China Geodetic Coordinate System 2000 (CGCS2000) and NEU systems, we first convert the data under the CGCS2000 system into the one under the space rectangular coordinate system (O-*XYZ*) and then convert it into the one under the NEU system.

Specifically, we assume that the position of the pedestrian under the CGCS2000 system is (L,B,H). Then, the position of the pedestrian under the space rectangular coordinate system can be described as:

(17) $$\left\{\begin{array}{c} X=(N+H)\cos B\cos L\\ Y=(N+H)\cos B\sin L\\ Z=\left[N(1-e^{2})+H)\right]\sin B \end{array}\right.$$

where *L*, *B* and *H* are the longitude, latitude and altitude of the pedestrian, respectively, under the CGCS2000 system, $N=a/\sqrt{1-e^{2}\sin^{2}B}$ is the prime vertical radius of the ellipsoid, $e=\sqrt{a^{2}-b^{2}}/a$ is the first eccentricity and a and b are the Earth semi-major axis and Earth semi-minor axis, which are set as 6378.137 km and 6356.7523142 km, respectively.

Therefore, the position of the pedestrian under the NEU system is:

(18) $$\left(\begin{array}{c} N_{QP}\\ E_{QP}\\ U_{QP} \end{array}\right)=\left(\begin{array}{ccc} -\sin B\cdot \cos L & -\sin B\cdot \sin L & \cos B\\ -\sin L & \cos L & 0\\ \cos B\cdot \cos L & \cos B\cdot \sin L & \sin B \end{array}\right)\left(\begin{array}{c} X_{Q}-X_{P}\\ Y_{Q}-Y_{P}\\ Z_{Q}-Z_{P} \end{array}\right)$$

where $\left(N_{QP},E_{QP},U_{QP}\right)$ is the coordinate under the NEU system of position Q relative to position P and $\left(X_{Q},Y_{Q},Z_{Q}\right)$ and $\left(X_{Q},Y_{Q},Z_{Q}\right)$ are the coordinates under the space rectangular coordinate system of P and Q in three different directions.

And the translation from BFS to NEU is shown as follow:

(19) $$\left[\begin{array}{ccc} N & E & U \end{array}\right]=C_{b}^{n}{\left[\begin{array}{ccc} X_{b} & Y_{b} & Z_{b} \end{array}\right]}^{T}$$

where $\left(X_{b},Y_{b},Z_{b}\right)$ is the position in BFS, (N,E,U) is the position in NEU, and the matrix $C_{b}^{n}$ describes the rotation relationship of the data under the BFS and NEU systems, as shown in Equation (20).

(20) $$C_{b}^{n}=\left(\begin{array}{ccc} \cos\gamma \sin\psi -\sin\gamma \sin\theta \sin\psi & \sin\gamma \sin\theta \cos\psi +\cos\gamma \sin\psi & -\sin\gamma \cos\theta \\ -\cos\theta \sin\psi & \cos\theta \cos\psi & \sin\theta \\ \cos\gamma \sin\theta sin\psi +\sin\gamma \cos\psi & -\cos\gamma \sin\theta \cos\psi +\sin\gamma \sin\psi & \cos\gamma \cos\theta \end{array}\right)$$

#### 3.2.2. Time Synchronization

**Figure 7:**
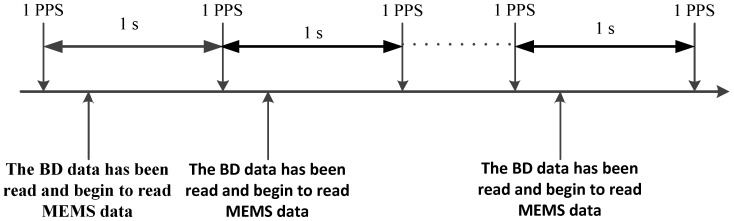
Process of time synchronization of the BD and MEMS. PPS: Pulse Per Second.

Since the BD receiver outputs the ephemeris data with a Pulse Per Second (PPS), we rely on the Advanced RISC Machines (ARM) external interrupt to conduct the pulse detection. When a PPS is detected, the ARM begins to read the data from the BD and then the one from the MEMS. When the next PPS is detected, the ARM switches to read the data from the BD again and then the one from the MEMS. This process guarantees the highly precise time synchronization of the BD and MEMS, as shown in Figure 7.

### 3.3. Tightly-Coupled Navigation System

#### 3.3.1. System State Equation

Since the positioning error at the current timestamp significantly relies on the ones at the previous timestamps, the positioning error at the k+1 timestamp can be calculated by:

(21) $$\left\{\begin{array}{c} \delta E\left(k+1\right)=\delta E\left(k\right)+\delta V_{E}\left(k\right)\sin\varphi +\varepsilon_{E}\\ \delta N\left(k+1\right)=\delta N\left(k\right)+\delta V_{N}\left(k\right)\cos\varphi +\varepsilon_{N}\\ \delta h\left(k+1\right)=\delta h\left(k\right)+\delta V_{h}\left(k\right)+\varepsilon_{h} \end{array}\right.$$

where δE, δN and δh are the positioning errors and $\varepsilon_{E}$, $\varepsilon_{N}$ and $\varepsilon_{h}$ are the corresponding noise under the NEU system in three different directions. $\delta V_{E}$, $\delta V_{N}$ and $\delta V_{h}$ are the velocity errors under the NEU system in three different directions, which can be calculated by:

(22) $$\left\{\begin{array}{c} \delta V_{E}\left(k+1\right)=\delta V_{E}\left(k\right)+\varepsilon_{V_{E}}\\ \delta V_{N}\left(k+1\right)=\delta V_{N}\left(k\right)+\varepsilon_{V_{N}}\\ \delta V_{h}\left(k+1\right)=\delta V_{h}\left(k\right)+\varepsilon_{V_{h}} \end{array}\right.$$

where $\varepsilon_{V_{E}}$, $\varepsilon_{V_{N}}$ and $\varepsilon_{V_{h}}$ are the noise under the NEU system in three different directions.

In the BD system, since there is a time offset between the satellite and receiver, we name the estimated range and the corresponding range rate between the satellite and receiver as the pseudo-range and pseudo-range rate, respectively. Based on this, we can find that the errors of the pseudo-range and pseudo-range rate mainly come from the equivalent clock error $\delta t_{u}$ and equivalent clock frequency error $\delta t_{ru}$. The pseudo-range and pseudo-range rate are calculated by:

(23) $$\left\{\begin{array}{c} \rho_{B}=\rho -\delta t_{u}-v_{\rho }\\ {\dot{\rho }}_{B}=\dot{\rho }-\delta t_{ru}-v_{\dot{\rho }} \end{array}\right.$$

where $\rho_{B}$ and ${\dot{\rho }}_{B}$ are the pseudo-range and pseudo-range rate, *ρ* and $\dot{\rho }$ are the real range and real range rate, $v_{\rho }$ and $v_{\dot{\rho }}$ are the Gaussian noise and $\delta t_{u}$ and $\delta t_{ru}$ satisfy the relations below.

(24) $$\left\{\begin{array}{c} \delta {\dot{t}}_{u}=\delta t_{ru}+\varepsilon_{u}\\ \delta {\dot{t}}_{ru}=-\beta_{ru}\delta t_{ru}+\varepsilon_{ru} \end{array}\right.$$

where $\varepsilon_{u}$ and $\varepsilon_{ru}$ are the Gaussian noise and *τ* is the correlation time.

Then, we select the positioning errors, δE,δN and δh, velocity errors, $\delta V_{E}$, $\delta V_{N}$ and $\delta V_{h}$, equivalent clock error, $\delta t_{u}$, and equivalent clock frequency error, $\delta t_{ru}$, to form the vector of system state variables in Equation (25).

(25) $$X={\left[\begin{array}{cccccccc} \delta E & \delta N & \delta h & \delta V_{E} & \delta V_{N} & \delta V_{h} & \delta t_{u} & \delta t_{ru} \end{array}\right]}^{T}$$

Finally, we construct the system state equation as follows.

(26) $$\begin{array}{c} X_{k+1}=\Phi_{k+1,k}X_{k}+\varepsilon_{k}\\ =\left[\begin{array}{cccccccc} 1 & 0 & 0 & T & 0 & 0 & 0 & 0\\ 0 & 1 & 0 & 0 & T & 0 & 0 & 0\\ 0 & 0 & 1 & 0 & 0 & T & 0 & 0\\ 0 & 0 & 0 & 1 & 0 & 0 & 0 & 0\\ 0 & 0 & 0 & 0 & 1 & 0 & 0 & 0\\ 0 & 0 & 0 & 0 & 0 & 1 & 0 & 0\\ 0 & 0 & 0 & 0 & 0 & 0 & 1 & T\\ 0 & 0 & 0 & 0 & 0 & 0 & 0 & 1-\beta_{tru}T \end{array}\right]X\left(k\right)+\left[\begin{array}{c} \varepsilon_{E}\\ \varepsilon_{N}\\ \varepsilon_{h}\\ \varepsilon_{V_{E}}\\ \varepsilon_{V_{N}}\\ \varepsilon_{V_{h}}\\ \varepsilon_{u}\\ \varepsilon_{ru} \end{array}\right] \end{array}$$

#### 3.3.2. System Observation Equation

By assuming that the location of the *i*-th satellite is $\left(x_{si},y_{si},z_{si}\right)$ and the location of the pedestrian, which is estimated by PDR, is $\left(x_{I},y_{I},z_{I}\right)$, we can calculate the pseudo-range by:

(27) $$\rho_{Ii}=\sqrt{{\left(x_{I}-x_{si}\right)}^{2}+{\left(y_{I}-y_{si}\right)}^{2}+{\left(z_{I}-z_{si}\right)}^{2}}$$

In addition, by setting (δx,δy,δz) as the vector of errors between $\left(x_{I},y_{I},z_{I}\right)$ and (x,y,z), where (x,y,z) is the real location of the pedestrian, $\left(x_{I},y_{I},z_{I}\right)$ can be described as:

(28) $$x_{I}=x+\delta x,y_{I}=y+\delta y,z_{I}=z+\delta z$$

Then, by applying the Taylor series expansion for Equation (27) at the point $\left(x-x_{si},y-y_{si},z-z_{si}\right)$, we can obtain:

(29) $$\rho_{Ii}=\sqrt{{\left(x-x_{si}\right)}^{2}+{\left(y-y_{si}\right)}^{2}+{\left(z-z_{si}\right)}^{2}}+\frac{\partial \rho_{Ii}}{\partial x_{Ii}}\delta x+\frac{\partial \rho_{Ii}}{\partial y_{Ii}}\delta y+\frac{\partial \rho_{Ii}}{\partial z_{Ii}}\delta z$$

where $\frac{\partial \rho_{Ii}}{\partial x_{Ii}}=\frac{x-x_{si}}{\sqrt{{\left(x-x_{si}\right)}^{2}+{\left(y-y_{si}\right)}^{2}+{\left(z-z_{si}\right)}^{2}}}=\frac{x-x_{si}}{r_{i}}=e_{i1}$, $\frac{\partial \rho_{Ii}}{\partial y_{Ii}}=\frac{y-y_{si}}{\sqrt{{\left(x-x_{si}\right)}^{2}+{\left(y-y_{si}\right)}^{2}+{\left(z-z_{si}\right)}^{2}}}=\frac{y-y_{si}}{r_{i}}=e_{i2}$ and $\frac{\partial \rho_{Ii}}{\partial z_{Ii}}=\frac{z-z_{si}}{\sqrt{{\left(x-x_{si}\right)}^{2}+{\left(y-y_{si}\right)}^{2}+{\left(z-z_{si}\right)}^{2}}}=\frac{z-z_{si}}{r_{i}}=e_{i3}$ and $r_{i}$ is the same as *ρ* in Equation (23), which presents the real distance between the i−th satellite and receiver.

Therefore, the pseudo-range equals:

(30) $$\rho_{Ii}=r_{i}+e_{i1}\delta x+e_{i2}\delta y+e_{i3}\delta z$$

Based on Equations (23) and (30), we obtain the observation equation of the pseudo-range as follows:

(31) $$Z_{\rho }=\left[\begin{array}{c} \rho_{I1}-\rho_{B1}\\ \rho_{I2}-\rho_{B2}\\ \rho_{I3}-\rho_{B3}\\ \rho_{I4}-\rho_{B4} \end{array}\right]=\left[\begin{array}{cccc} e_{11} & e_{12} & e_{13} & -1\\ e_{21} & e_{22} & e_{23} & -1\\ e_{31} & e_{32} & e_{33} & -1\\ e_{41} & e_{42} & e_{43} & -1 \end{array}\right]\left[\begin{array}{c} \delta x\\ \delta y\\ \delta z\\ \delta t_{u} \end{array}\right]-\left[\begin{array}{c} v_{\rho 1}\\ v_{\rho 2}\\ v_{\rho 3}\\ v_{\rho 4} \end{array}\right]$$

According to the relationship of the data under the NEU and O-*XYZ* systems, (δx,δy,δz) can be calculated by:

(32) $$\left\{\begin{array}{c} \delta x=-\sin\lambda \delta E-\sin L\cos\lambda \delta N+\cos L\cos\lambda \delta h\\ \delta y=\cos\lambda \delta E-\sin L\sin\lambda \delta N+\cos L\sin\lambda \delta h\\ \delta z=\cos L\delta N+\sin L\delta h \end{array}\right.$$

where *L* and *λ* represent the longitude and latitude, and (δE,δN,δh) represents the vector of position errors under the NEU system. We rewrite the observation equation of the pseudo-range as:

(33) $$Z_{\rho }\left(t\right)=H_{\rho }\left(t\right)X\left(t\right)+V_{\rho }\left(t\right)$$

where:

(34) $$H_{\rho }\left(t\right)=\left[\begin{array}{cccc} h_{\rho 1} & 0_{4\times 3} & h_{\rho 2} & 0_{4\times 1} \end{array}\right]$$

(35) $$h_{\rho 1}=\left[\begin{array}{ccc} a_{11} & a_{12} & a_{13}\\ a_{21} & a_{22} & a_{23}\\ a_{31} & a_{32} & a_{33}\\ a_{41} & a_{42} & a_{43} \end{array}\right],h_{\rho 2}=\left[\begin{array}{c} -1\\ -1\\ -1\\ -1 \end{array}\right]$$

(36) $$\left\{\begin{array}{c} a_{i1}=-e_{i1}\sin\lambda +e_{i2}\cos\lambda \\ a_{i2}=-e_{i1}\sin L\cos\lambda -e_{i2}\sin L\sin\lambda +e_{i3}\cos L\\ a_{i3}=e_{i1}\cos L\cos\lambda +e_{i2}\cos L\sin\lambda +e_{i3}\sin L \end{array}\right.$$

and:

(37) $$V_{\rho }\left(t\right)=\left[\begin{array}{c} V_{\rho 1}\\ V_{\rho 2}\\ V_{\rho 3}\\ V_{\rho 4} \end{array}\right]$$

Similarly, we can obtain the observation equation of pseudo-range rate in Equation (38).

(38) $$Z_{\rho }=\left[\begin{array}{c} {\dot{\rho }}_{I1}-{\dot{\rho }}_{B1}\\ {\dot{\rho }}_{I2}-{\dot{\rho }}_{B2}\\ {\dot{\rho }}_{I3}-{\dot{\rho }}_{B3}\\ {\dot{\rho }}_{I4}-{\dot{\rho }}_{B4} \end{array}\right]=\left[\begin{array}{cccc} e_{11} & e_{12} & e_{13} & -1\\ e_{21} & e_{22} & e_{23} & -1\\ e_{31} & e_{32} & e_{33} & -1\\ e_{41} & e_{42} & e_{43} & -1 \end{array}\right]\left[\begin{array}{c} \dot{\delta }x\\ \dot{\delta }y\\ \dot{\delta }z\\ \dot{\delta }t_{ru} \end{array}\right]-\left[\begin{array}{c} v_{\dot{\rho }1}\\ v_{\dot{\rho }2}\\ v_{\dot{\rho }3}\\ v_{\dot{\rho }4} \end{array}\right]$$

where $\dot{\delta }x$, $\dot{\delta }y$ and $\dot{\delta }z$ are the velocity errors under the O-XYZ system in three different directions, which can be easily converted into the ones under the NEU system based on the relations in Equation (39).

(39) $$\left\{\begin{array}{c} \dot{\delta }x=-\sin\lambda \delta V_{E}-\sin L\cos\lambda \delta V_{N}+\cos L\cos\lambda \delta V_{h}\\ \dot{\delta }y=\cos\lambda \delta V_{E}-\sin L\sin\lambda \delta V_{N}+\cos L\sin\lambda \delta V_{h}\\ \dot{\delta }z=\cos L\delta V_{N}+\sin L\delta V_{h} \end{array}\right.$$

where $\left(\delta V_{E},\delta V_{N},\delta V_{h}\right)$ represents the vector of velocity errors under the NEU system. Then, the observation equation of the pseudo-range rate can be rewritten as:

(40) $$Z_{\dot{\rho }}\left(t\right)=H_{\dot{\rho }}\left(t\right)X\left(t\right)+V_{\dot{\rho }}\left(t\right)$$

where:

(41) $$H_{\dot{\rho }}\left(t\right)=\left[\begin{array}{cccc} 0_{4\times 3} & h_{\dot{\rho }1} & 0_{4\times 1} & h_{\dot{\rho }2} \end{array}\right]$$

(42) $$h_{\dot{\rho }1}=\left[\begin{array}{ccc} b_{11} & b_{12} & b_{13}\\ b_{21} & b_{22} & b_{23}\\ b_{31} & b_{32} & b_{33}\\ b_{41} & b_{42} & b_{43} \end{array}\right],h_{\dot{\rho }2}=\left[\begin{array}{c} -1\\ -1\\ -1\\ -1 \end{array}\right]$$

(43) $$\left\{\begin{array}{c} b_{i1}=-e_{i1}\sin\lambda +e_{i2}\cos\lambda \\ b_{i2}=-e_{i1}\sin L\cos\lambda -e_{i2}\sin L\sin\lambda +e_{i3}\cos L\\ b_{i3}=e_{i1}\cos L\cos\lambda +e_{i2}\cos L\sin\lambda +e_{i3}\sin L \end{array}\right.$$

(44) $$V_{\dot{\rho }}\left(t\right)=\left[\begin{array}{c} V_{\dot{\rho }1}\\ V_{\dot{\rho }2}\\ V_{\dot{\rho }3}\\ V_{\dot{\rho }4} \end{array}\right]$$

Finally, we construct the system observation equation as follows.

(45) $$\left[\begin{array}{c} Z_{\rho }\\ Z_{\dot{\rho }} \end{array}\right]=\left[\begin{array}{c} H_{\rho }\\ H_{\dot{\rho }} \end{array}\right]X+\left[\begin{array}{c} V_{\rho }\\ V_{\dot{\rho }} \end{array}\right]$$

#### 3.3.3. Positioning

After the system state equation and system observation equation are established, we rely on the EKF to modify the position errors, δN and δE, for the position tracking model in Equation (46).

(46) $$\left\{\begin{array}{c} N_{k}=N_{k-1}+d_{k-1}\cos\theta_{k-1}+\delta N_{k}\\ E_{k}=E_{k-1}+d_{k-1}\sin\theta_{k-1}+\delta E_{k} \end{array}\right.$$

where $\left(E_{k-1},N_{k-1}\right)$, $d_{k-1}$ and $\theta_{k-1}$ are the coordinate, stride length and heading value of the previous position, and $\left(E_{k},N_{k}\right)$ is the current estimated position.

## 4. Experimental Results

**Figure 8:**
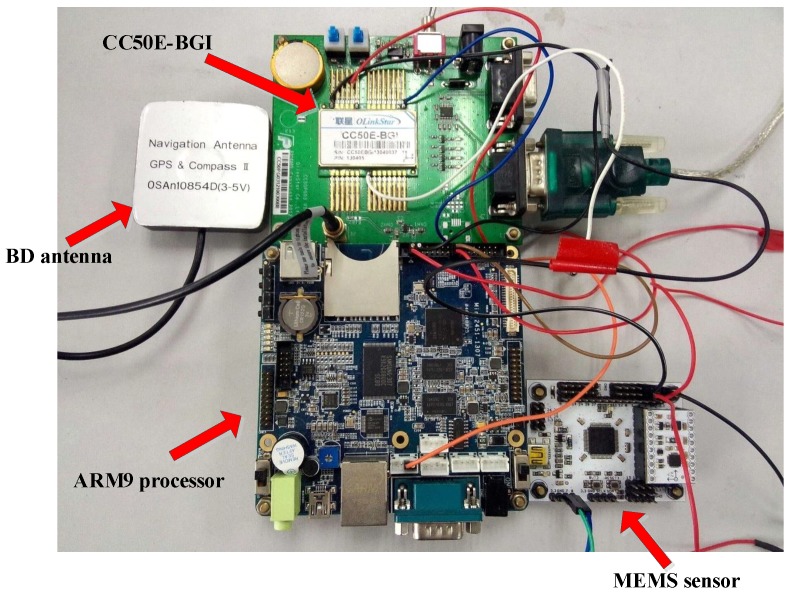
Hardware platform.

**Figure 9:**
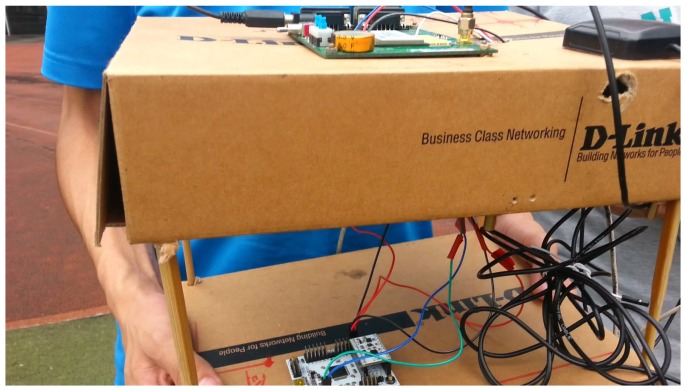
The way to hold the prototype device.

The hardware platform for the experiments is shown in Figure 8. We select the CC50E-BGI (OLinkStar) as the receiver, which can be used to record the ephemeris, PPS, pseudo-range and pseudo-range rate of the BD. The MPU9150 9-axis MEMS sensor contains the HMC5883 magnetometer, the MPU6050 gyroscope and an accelerometer, and the sensors’ specifications are shown in Table 1. The MEMS sensor calibration is indeed necessary, such as installation error and zero drift. We did this in our previous works. The STM32F103RBT6 chip is selected as the control module with a sampling rate of 50 Hz. The data from the BD and MEMS sensor are processed by using the ARM9 processor. Additionally, during our test, the device is held by hand, as shown in Figure 9. The MEMS sensor is far away from the ARM9 processor and BD antenna, because the metallic materials of the two devices will interfere with the magnetometer.

### 4.1. Performance of Stride Length Estimation

As it is hard to measure every stride length, comparing with real length and the estimated one is impossible. To examine the performance of stride length estimation using the empirical model improved by the BP neural network, we invited three volunteers with different height, 153, 170 and 183 cm, to walk along the same straight path of 100 meters. The total walking length is estimated using the presented method, as well as using a fixed stride length. During the testing, the volunteers are required to walk with three different patterns, walking slowly, walking normally and walking quickly. The positioning results under different scenarios are compared in Table 2.

From Table 2, we can observe that the positioning errors of PDR using the BP neural network are much smaller than the ones achieved by the PDR with fixed stride length. This means that the presented method can estimate stride length accurately. It can be found that for a height of 170 cm, the walking normally pattern PDR with a fixed length works better than using the presented method, but other conditions are opposite. PDR based on the presented method is applicable for different people and walking speeds, but PDR with a fixed stride length just performs well in some conditions.

### 4.2. Positioning in a Playground Surrounded by Trees

**Figure 10:**
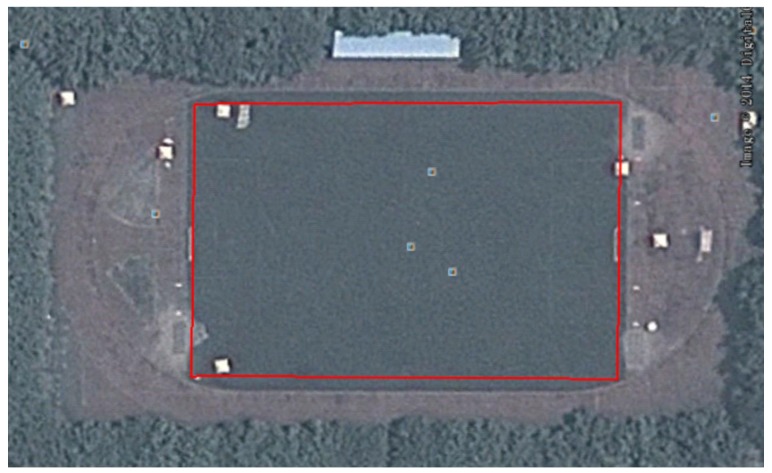
Playground surrounded by trees.

**Figure 11:**
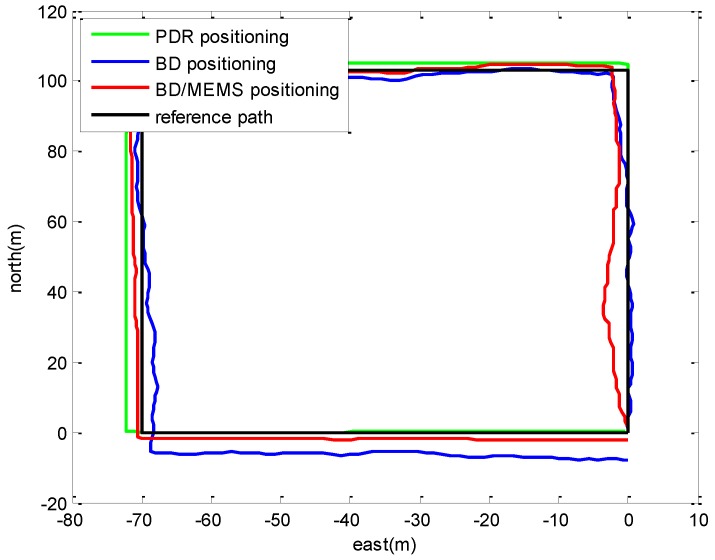
Comparison of the trajectories.

The layout of the playground surrounded by trees is shown in Figure 10. A volunteer holding the BD/MEMS receiver walked along a rectangular path (marked in red) with a length and width of 103 and 70 m, respectively. Figure 11 compares the trajectories of the proposed BD/MEMS positioning, PDR positioning and BD positioning, respectively. The comparison of the positioning errors of different positioning methods is shown in Table 3.

From Table 3, we can observe that the Root Mean Square (RMS) errors of the BD/MEMS tightly-coupled system are smaller than the PDR and BD system. The proposed system performs well in the playground surrounded by trees.

### 4.3. Positioning in a Thick Foliage Environment

**Figure 12:**
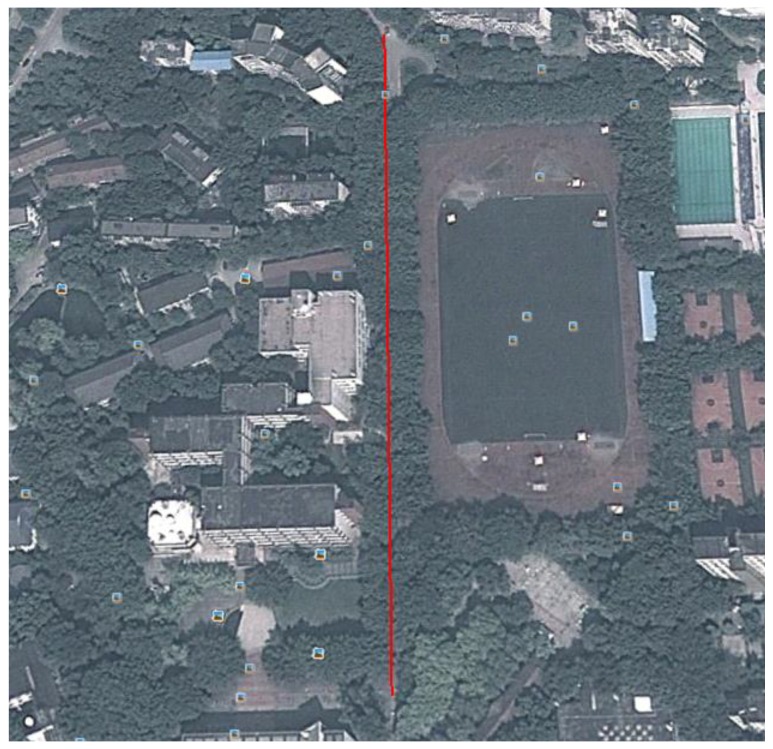
Thick foliage environment.

Figure 12 shows the layout of the thick foliage environment. In this environment, a volunteer holding the BD/MEMS receiver walked along a straight path (marked in red) with a length of 290 m. There are no visible satellites, and the satellite signal is severely degraded.

**Figure 13:**
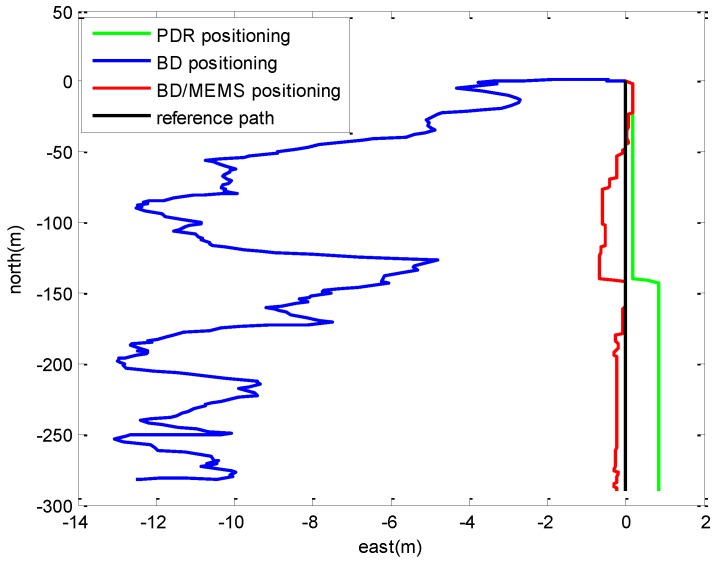
Comparison of trajectories.

Figure 13 compares the trajectories estimated by the proposed BD/MEMS positioning, PDR positioning and BD positioning, respectively. Since the satellite signal is seriously degraded in the thick foliage environment, the performance of the BD system is the poorest. The PDR system performs well at the beginning of the walking, but its performance deteriorates with time due to the problem of error accumulation. On the contrary, the proposed system always performs well and stably. The comparison of positioning errors of different positioning methods is shown in Table 4.

### 4.4. Positioning in an Open Sky Environment for a Long Time

**Figure 14:**
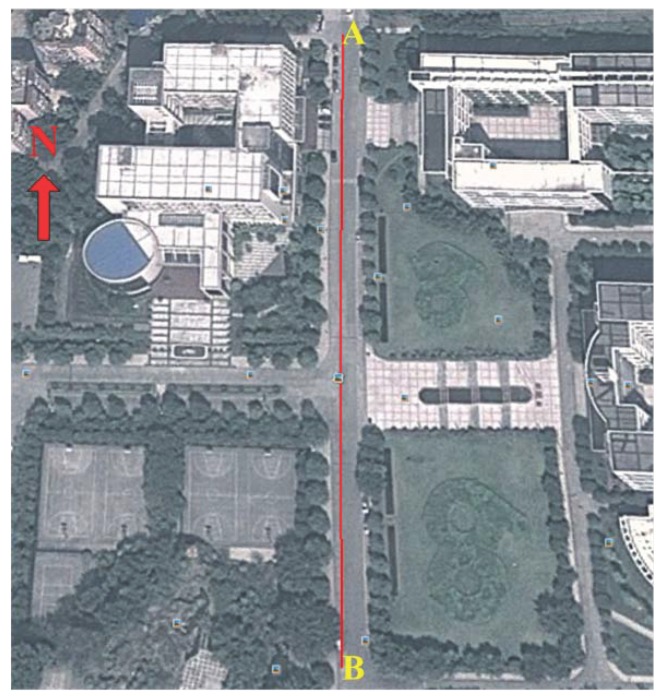
Open sky environment.

We continue to perform the testing in an open sky environment with a long period of time, as shown in Figure 14. The length of the path is about 230 m. A volunteer was invited to hold the BD/MEMS receiver and walked along this path for three rounds. It took about 25 min.

**Figure 15:**
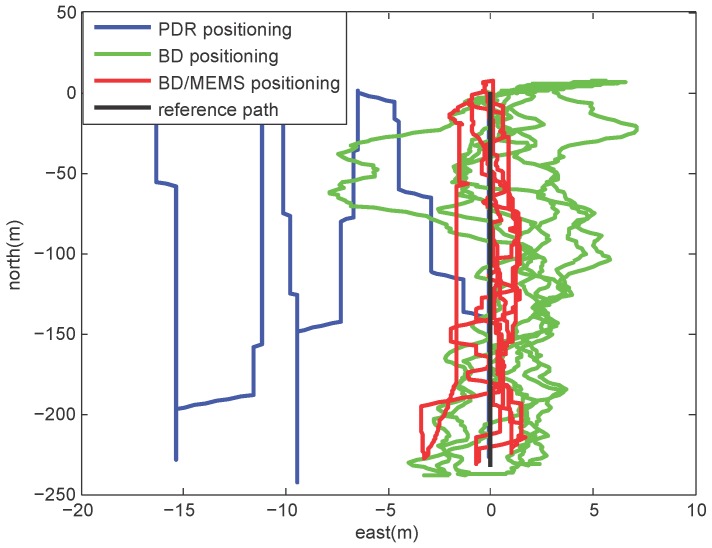
Comparison of trajectories.

Figure 15 compares the trajectories estimated by using the proposed BD/MEMS positioning, PDR positioning and BD positioning, respectively. From this figure, we can find that the trajectory of PDR trajectory deviates from the reference trajectory gradually, whereas the proposed system is able to overcome the problem of error accumulation, as well as to construct the precise trajectory. To show this result more clearly, we compare the positioning errors of different positioning methods in Table 5.

## 5. Conclusions

This paper proposed a low-cost BD/MEMS tightly-coupled system for pedestrian navigation and built a prototype. Experiments were done in different environments, such as the playground surrounded by trees, the thick foliage environment and the open sky environment. The results demonstrate that the designed BD/MEMS tightly-coupled system outperforms the PDR and BD systems from the comparisons of the trajectories and positioning errors. However, using carrier phase measurement, differential positioning and a particle filter to further improve the positioning accuracy of our system forms an interesting future work.

## Figures and Tables

**Table 1:** Specifications of MPU9150.

Patterns	Accelerometer		Gyroscope		Magnetometer
	Full Scale Range (g)	Sensitivity (LSB/mg)	Full Scale Range (g)	Sensitivity (LSB/mg)	Full Scale Range (g)
1	±2	16,384	±250	131	±1200
2	±4	8192	±500	65.5	
3	±6	4096	±1000	32.8	
4	±16	2048	±2000	16.4	

**Table 2:** Positioning errors with different height and under different walking models.

Height	Models					
	Walking Slowly		Walking Normally		Walking Quickly	
	Adaptive Stride Length	Fixed Length	Adaptive Stride Length	Fixed Length	Adaptive Stride Length	Fixed Length
153 cm	0.8 m	3.8 m	0.7 m	3.5 m	6.4 m	12.3 m
170 cm	7.3 m	7.9 m	6.9 m	5.0 m	6.0 m	12.2 m
183 cm	1.0 m	8.5 m	10.5 m	18.5 m	4.8 m	19.5 m

**Table 3:** Comparison of positioning errors.

Different Positioning Techonologies	RMS Errors in East (m)	RMS Errors in North (m)	RMS Errors (m)
BD/MEMS	1.2290	0.9787	1.5710
PDR	1.2965	1.1766	1.7508
BD	0.8786	3.9074	4.0050

**Table 4:** Comparison of positioning errors.

Different Positioning Techonologies	RMS Errors in East (m)	RMS Errors in North (m)	RMS Errors (m)
BD/MEMS	0.3402	0.8676	0.9302
PDR	0.6123	0.7403	0.9607
BD	9.7366	5.2195	11.0481

**Table 5:** Comparison of positioning errors.

Different Positioning Techonologies	RMS Errors in East (m)	RMS Errors in North (m)	RMS Errors (m)
BD/MEMS	1.0545	1.2258	1.6854
PDR	8.0707	5.0884	9.8844
BD	2.8146	3.0804	5.5297

## References

[1] Kupper A. (2005). Architectures and protocols for location services. Location-Based Services: Fundamentals and Operation.

[2] Zhen J., Jie W., Sabatino P. GUI: GPS-Less traffic congestion avoidance in urban areas with inter-vehicular communication. Proceedings of the 11th International Conference on Mobile Ad Hoc and Sensor Systems (MASS).

[3] Flores G., Zhou S., Lozano R., Castillo P. A vision and GPS-based real-time trajectory planning for MAV in unknown urban environments. Proceedings of the International Conference on Unmanned Aircraft Systems (ICUAS).

[4] Mubarak O.M., Dempster A.G. Beidou: A GPS alternative for Pakistan’s naval vessels. Proceedings of the 10th International Bhurban Conference on Applied Sciences and Technology (IBCAST).

[5] Rabaoui A., Viandier N., Duflos E., Marais J., Vanheeghe P. (2012). Dirichlet process mixtures for density estimation in dynamic nonlinear modeling: application to GPS positioning in urban canyons. Signal Process..

[6] Fang Z., Huang H. (2010). Application of MEMS technology in military purpose equipment. ElectroMech. Eng..

[7] Yu L., Gao Z., Li D. Hybrid particle filter for vehicle MEMS-INS. Proceedings of the 2nd International Conference on Advanced Computer Control (ICACC).

[8] Hassansin M.A., Taha M.M.R., Noureldin A. (2004). Automization of an INS/GPS intecrated system using genetic optimization. Autom. Congr..

[9] Niu X., Ban Y., Zhang Q., Zhang T., Zhang H., Liu J. (2015). Quantitative Analysis to the Impacts of IMU Quality in GPS/INS Deep Integration. Micromachines.

[10] Schmidt P., Thoelert S., Furthner J., Meurer M. Signal in space (SIS) analysis of new GNSS satellites. Proceedings of the Satellite Navigation Technologies and European Workshop on GNSS Signals and Signal Processing (NAVITEC).

[11] Liang G., Li F., Zhang X., Chen Y. Characteristic and anti-jamming performance of new generation GNSS signals. Proceedings of the 2012 IEEE 14th International Conference on Communication Technology (ICCT).

[12] Kong S. (2015). SDHT for fast detection of weak GNSS signals. Sel. Areas Commun..

[13] Montillet J.P., Yu K. Modified leaky LMS algorithms applied to satellite positioning. Proceedings of the Vehicular Technology Conference.

[14] Li X., Dick G., Lu C., Ge M., Nilsson T., Ning T., Wickert J., Schuh H. (2015). Multi-GNSS meteorology: Real-time retrieving of atmospheric water vapor from BeiDou, Galileo, GLONASS, and GPS observations. Geosci. Remote Sens..

[15] Perlmutter M., Robin L. High-performance, low cost inertial MEMS: A market in motion. Proceedings of the Position Location and Navigation Symposium (PLANS).

[16] Vitanov I., Aouf N. Fault diagnosis for MEMS INS using unscented Kalman filter enhanced by Gaussian process adaptation. Proceedings of the Adaptive Hardware and Systems (AHS).

[17] Aggarwal P., Syed Z., El-Sheimy N. Thermal calibration of low cost MEMS sensors for land vehicle navigation system. Proceedings of the Vehicular Technology Conference.

[18] Akeila E., Salcic Z., Swain A. (2014). Reducing low-cost INS error accumulation in distance estimation using self-resetting. Instrum. Measur..

[19] Angrisano A. (2010). GNSS/INS Integration Methods. Ph.D. Thesis.

[20] Zhuang Y., Chang H., El-Sheimy N. (2013). A MEMS multi-sensors system for pedestrian navigation. China Satellite Navigation Conference (CSNC) 2013 Proceedings.

[21] Jia R., Wu C., Zhi Q., Yu B. Research on lower cost MEMS IMU and GPS integrating algorithm. Proceedings of the 4th China Satellite Navigation Conference.

[22] Lachapelle G., Godha S., Cannon M. Performance of integrated HSGPS-IMU technology for pedestrian navigation under signal masking. Proceedings of the European navigation conference.

[23] Akca T., Demirekler M. An adaptive unscented Kalman filter for tightly coupled INS/GPS integration. Proceedings of the Position Location and Navigation Symposium (PLANS).

[24] Clark B.J., Bevly D.M. GPS/INS integration with fault detection and exclusion in shadowed environments. Proceedings of the 2008 IEEE/ION Position Location and Navigation Symposium (PLANS).

[25] Chu H., Tsai G., Chiang K., Duong T. (2013). GPS/MEMS INS data fusion and map matching in urban areas. Sensors.

[26] Godha S., Lachapelle G., Cannon M.E. Integrated GPS/INS system for pedestrian navigation in a signal degraded environment. Proceedings of the ION GNSS 2006.

[27] Godha S., Petovello M.G., Lachapelle G. Performance analysis of MEMS IMU/HSGPS/magnetic sensor integrated system in urban canyons. Proceedings of the ION GNSS 2005.

[28] O’Keefe K., Jiang Y., Petovello M. An investigation of tightly-coupled UWB/low-cost GPS for vehicle-to-infrastructure relative positioning. Proceedings of the 2014 IEEE Radar Conference.

[29] Weinberg H. (2002). Using the ADXL202 in pedometer and personal navigation applications. Analog Devices AN-602 Appl. Note.

[30] Alvarez D., Gonzalez R.C., Lopez A., Alvarez J.C. Comparison of step length estimators from wearable accelerometer devices. Proceedings of the 28th Annual International Conference of the IEEE, Engineering in Medicine and Biology Society.

[31] Shin B., Lee J.H., Lee H. Indoor 3D pedestrian tracking algorithm based on PDR using smarthphone. Proceedings of the 2012 12th International Conference on Control, Automation and Systems (ICCAS).

[32] Liu J., Zhu B. Application of BP neural network based on GA in function fitting. Proceedings of the 2012 2nd International Conference on Computer Science and Network Technology (ICCSNT).

[33] Valenti R.G., Dryanovski I., Xiao J. (2016). A Linear Kalman Filter for MARG Orientation Estimation Using the Algebraic Quaternion Algorithm. Instrum. Measur..

